# Molecular detection of transcriptionally active ovine papillomaviruses in commercial equine semen

**DOI:** 10.3389/fvets.2024.1427370

**Published:** 2024-07-03

**Authors:** Anna Cutarelli, Francesca De Falco, Roberta Brunetti, Michele Napoletano, Giovanna Fusco, Sante Roperto

**Affiliations:** ^1^Istituto Zooprofilattico Sperimentale del Mezzogiorno, Naples, Italy; ^2^Dipartimento di Medicina Veterinaria e delle Produzioni Animali, Università degli Studi di Napoli Federico II, Naples, Italy; ^3^Area Science Park, Campus di Baronissi, Università degli Studi di Salerno, Salerno, Italy

**Keywords:** ddPCR, ovine papillomaviruses, pregnancy loss, semen, stallions

## Abstract

Virological evaluation was performed on equine semen to detect the presence of papillomaviruses (PVs) using droplet digital polymerase chain reaction (ddPCR) as the aim of this study was to investigate whether the sperm from asymptomatic stallions harbors ovine papillomaviruses (OaPVs). Twenty-seven semen samples were analyzed, 18 of which were commercially acquired. The remaining nine samples comprising semen and peripheral blood, were collected from nine stallions with no apparent signs of PV-related diseases during clinical examination at the Didactic Veterinary University Hospital (DVUH) of Naples. OaPV was detected in 26 semen samples. OaPV1 was the most prevalent virus infecting equine semen. OaPV1 infected 21 semen samples (~80.8%) and showed a high number of DNA and RNA copies per microliter. qPCR was used to detect OaPV1 DNA in the 18 semen samples. ddPCR was used to detect and quantify the expression of OaPV2, OaPV3, and OaPV4. qPCR failed to detect DNA for these genotypes. Additionally, ddPCR was used to detect the transcriptionally active OaPV1 in six blood and semen samples from the same stallion. ddPCR failed to detect any nucleic acids in OaPVs in peripheral blood samples from the three stallions. In one semen sample, ddPCR detected OaPV1 DNA but failed to detect any nucleic acid in the remaining two semen samples, and peripheral blood from the same animals of the remaining 18 semen samples was not available, OaPV1 and OaPV4 were responsible for nine and five single infections, respectively. No single infections with either OaPV3 or OaPV4 were seen.

## 1 Introduction

Papillomaviruses (PVs) are small, non-enveloped, epitheliotropic, double-stranded DNA viruses that infect the cutaneous and mucosal epithelia of a wide variety of mammals and vertebrates (1).

Ovine papillomaviruses (OaPVs, *Ovis aries* papillomaviruses) are oncogenic viruses with four genotypes (2). OaPV1, OaPV2, and OaPV4 belong to the genus *Deltapapillomavirus*, whereas OaPV3 belongs to *Dyokappapapillomavirus* (3). OaPV1, OaPV2, and OaPV4 infect both keratinocytes and fibroblasts (4). They have also been associated with cutaneous papillomas and fibropapillomas in sheep (5, 6). They display their transforming activity via the E5 oncoprotein, which is the most highly conserved oncoprotein among delta-papillomaviruses (7, 8). OaPV3 is believed to be an epitheliotropic virus that has been associated with squamous cell carcinomas of ovine skin because it possesses transforming properties via E6 and E7 oncoproteins (9, 10). Recent studies have raised questions regarding actual OaPV3 tropism. OaPV3 has been found to be transcriptionally active in the peripheral blood of apparently healthy cattle as well as in both epithelial and mesenchymal tumors of bovine bladders (11, 12). It is likely that OaPV3 has both mesenchymal and epithelial tropism, similar to ovine fibropapillomaviruses.

PVs slowly co-evolve with their respective hosts, showing minimal cross-transfer between species (13). However, OaPV2 DNA sequences have been detected in a sarcoid-like mass in the mouth of a pig and in teat papillomatosis in hair goats (14, 15). OaPV DNA was first detected and quantified in equine sarcoid samples (16). OaPV DNA and mRNA have been detected in cattle (11, 12). These studies provide valuable insights into the mechanism by which OaPVs affect cross-species transmission and infection, similar to BPVs.

No epidemiological data on OaPV infections in equines are available. To better understand the epidemiology of PV infection in horses, we performed a virological evaluation of semen from asymptomatic stallions using droplet digital polymerase chain reaction (ddPCR). ddPCR is used to detect and directly quantify low-abundance pathogens with a high degree of sensitivity; therefore, ddPCR provides a more precise and reproducible detection of pathogen loads in the clinical diagnosis of infectious diseases, including viral diseases (17). ddPCR yields significantly fewer inaccurate false-negatives and positives than real-time qPCR in samples with low viral loads (18). Furthermore, ddPCR is currently the most accurate and sensitive molecular procedure for measuring the abundance of papillomavirus nucleic acids in both human and veterinary medicine (19, 20). Although PVs are mostly associated with cancer, recent studies have identified human papillomavirus (HPV) infections as a possible cause of impaired human reproduction (21). Growing evidence suggests that HPV semen infections may cause infertility (22). Therefore, it has been suggested that HPVs may be responsible for both cancer and subfertility (23).

Little information is available concerning the actual incidence of PV infections in male domestic animals, particularly regarding the presence and significance of PVs in semen. In addition to sporadic cases of equine papillomaviruses (EcPVs), BPVs have only been detected in the semen of healthy stallions so far (24).

This study aimed to utilize ddPCR to detect and quantify OaPV nucleic acids in the semen of healthy stallions.

## 2 Materials and methods

### 2.1 Sample collection and processing

We examined 27 equine semen samples, 18 of which were commercially obtained. Nine additional samples, composed of semen and peripheral blood samples, were collected from nine stallions with no apparent signs of PV-related disease during clinical examination at the Didactic Veterinary University Hospital (DVUH) of Naples. All animal studies were approved by the Institutional Animal Care and Use Committee, protocol PG/2024/0023599, Naples University Federico II.

### 2.2 DNA extraction

DNA was extracted from 27 semen samples and nine horse blood samples. DNA was extracted from semen as follows: 250 μL of sperm was added to 10 mL Buffer 1 150 mM NaCl, 10 mM EDTA (pH 8.0) and vortexed for 10 s at full speed. The mixture was centrifuged for 10 min at 4,000 rpm (2,500 × g) and the supernatant was carefully removed, leaving ~1 mL of pellet which was transferred to a 2.0 mL microcentrifuge tube. Buffer 1 (0.5 mL) was added and the mixture was centrifuged for 2 min at full speed in a microcentrifuge. The pellet was resuspended in 300 μL Buffer 2 [100 mM Tris·Cl (pH 8.0), 10 mM EDTA, 500 mM NaCl, 1% SDS, 2% ß-mercaptoethanol] and 100 μL of Proteinase K solution was added before incubation for 2 h at 55°C. Then, 400 μL Buffer AL and 400 μL ethanol were added to the sample and the mixture was vortexed. Step 5 of the tissue protocol was performed using a QIAamp DNA Mini Kit (Qiagen, Wilmington, DE, United States). DNA was extracted from blood samples using the DNeasy Blood & Tissue Kit (Qiagen, Wilmington, DE, United States) according to the manufacturer’s instructions.

### 2.3 Droplet digital polymerase chain reaction

For ddPCR (Bio-Rad Laboratories, Hercules, CA, United States), the QX100 ddPCR System was employed according to the manufacturer’s instructions. The reaction was performed in a final volume of 22 μL, consisting of 11 μL of ddPCR Supermix for Probes (2X; Bio-Rad Laboratories, Hercules, CA, United States), 0.9 μM primer, and 0.25 μM of probe with 7 μL sample DNA corresponding to 100 ng. The primer and probe sequences for OaPV (Table 1), as well as the tool to generate droplets and a thermal profile, have been described previously (20). Each sample was analyzed in triplicate to ensure accuracy. Samples with very few positive droplets were reanalyzed to ensure that these low-copy number samples were not cross-contaminated.

**Table 1:** Primers and probes used for detection of OaPVs in ddPCR and qPCR.

OaPV1	CCTGATTCTATGACTGTAAGAGGC	CTCCCCACAGAAGTCCAAG	TGCAACAGCAGAGTCCCATCAGAAG FAM	E5 5’UTR/ORF E5	119	(25)
OaPV2	AGTTCCCGCTCTGATTTACC	ATGGCGGACGTATACTTGTTC	ATTGCCAGCAGTCTCCTCAGTCATTC FAM	L1	134	
OaPV3	AGCCCACACTCCCTGATATAG	TTCAGTCTTTGACAGCACCTC	AGCAACCAGCACTGTACACGCTAT	E7	145	
OaPV4	GGGTTCTATGGTGTCTGCTTAG	GCTCAAAATGGTCTACTGTTGC	CAGGAATGCTCTGTGCAGGGTATAGTG FAM	E5	102	(25)

### 2.4 RNA extraction and one-step reverse transcription (RT)-ddPCR

RNA was extracted from the above samples using the RNeasy Plus Mini Kit (Qiagen, Hilden, Germany) according to the manufacturer’s instructions. This kit contains genomic DNA (gDNA) eliminator spin columns. One hundred nanograms of RNA (100 ng) was used with the One-Step RT-ddPCR Advanced Kit for Probes (Bio-Rad Laboratories, Hercules, CA, United States) according to the manufacturer’s instructions, as previously described (12). A one-step reaction was performed on samples in which reverse transcriptase (RT) was added (RT+), and in those without RT (RT-).

### 2.5 Statistical analysis

Fisher’s exact test was performed to evaluate the actual differences in the prevalence of the four papillomavirus genotypes in the same animals. McNemar’s test for two related binomial proportions (conditional) was used to evaluate the agreement between ddPCR and qPCR performed on the same animals. The statistical significance was set at *p* ≤ 0.05 for both tests. Statistical analyses were performed using RStudio^®^ software ver. 4.2 (RStudio, Boston, MA, United States). Following Biron et al. (2016), the sensitivity of the method was calculated as the number of semen samples positive for OaPV DNA divided by the total number of horses examined. According to Damerla et al. (26), specificity is defined as the number of negative samples with zero OaPV-positive droplets divided by the total number of negative samples.

## 3 Results

### 3.1 Virological assessment

**Figure 1:**
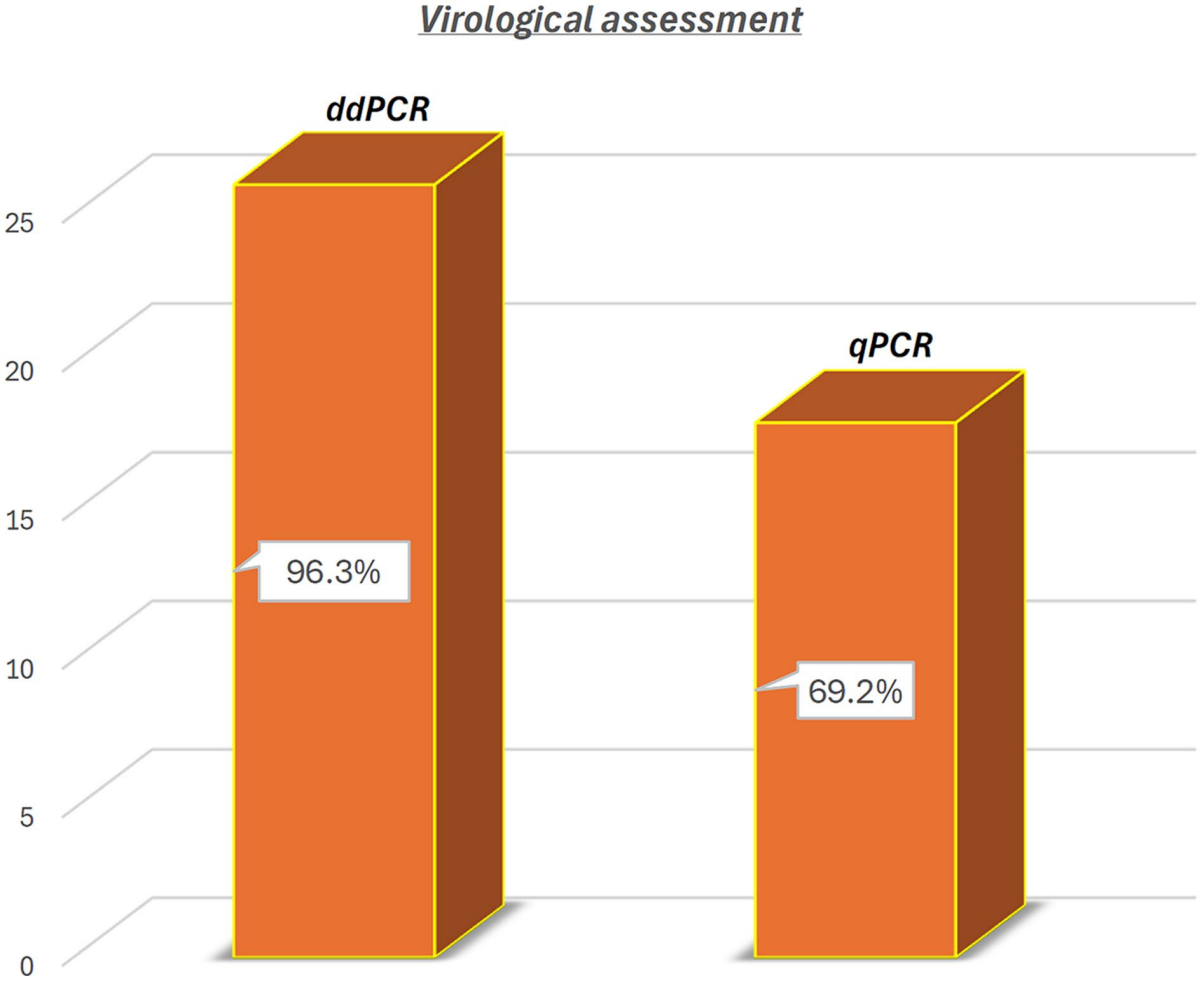
Detection of OaPV DNA in 26 (96.3%) and in 18 of 27 semen samples (69.2%) through ddPCR and qPCR, respectively. Differences were significant using McNemar test (*p* value ≤ 0.05).

**Figure 2:**
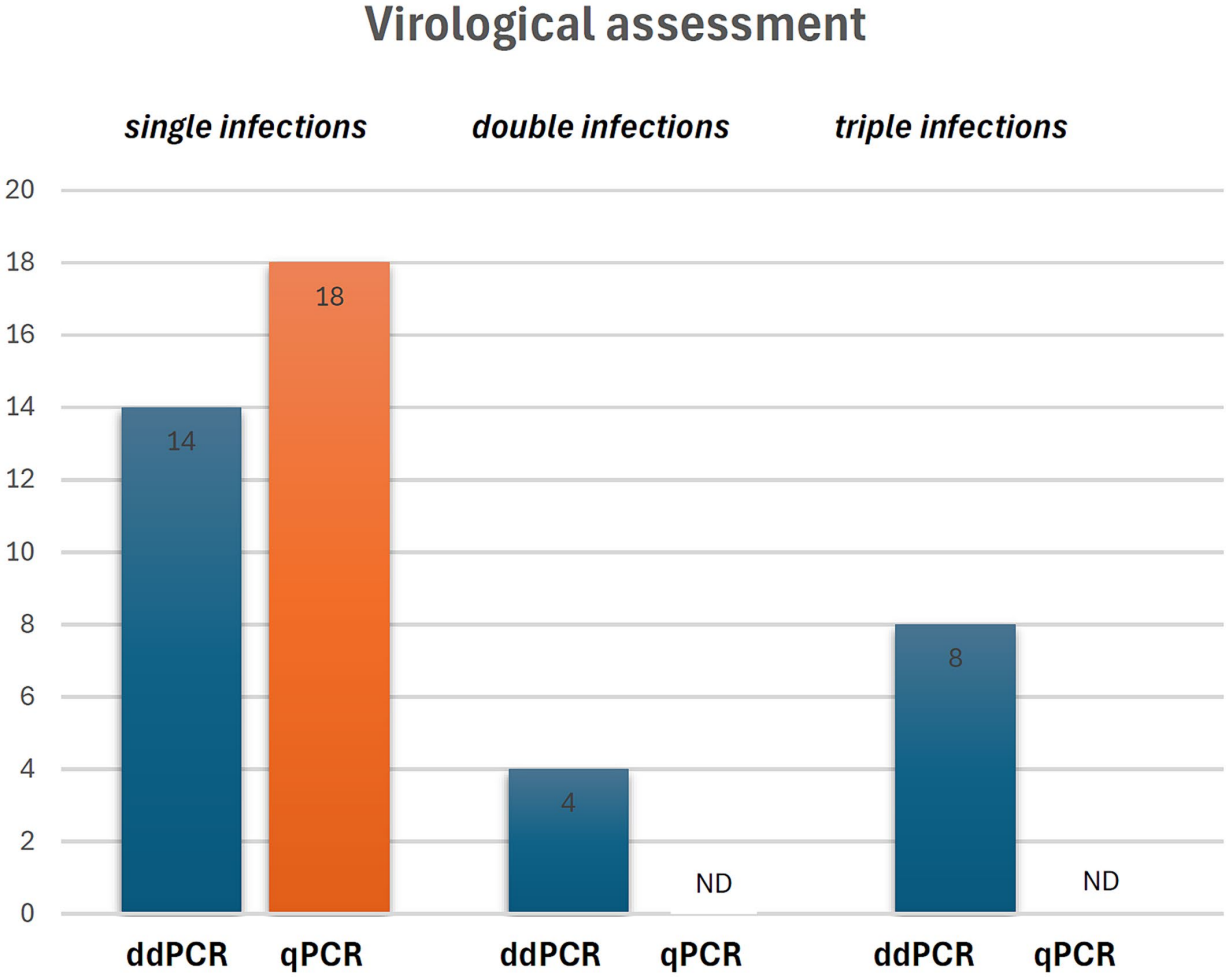
Single and multiple infection revealed by ddPCR and qPCR. ND, not detected.

**Figure 3:**
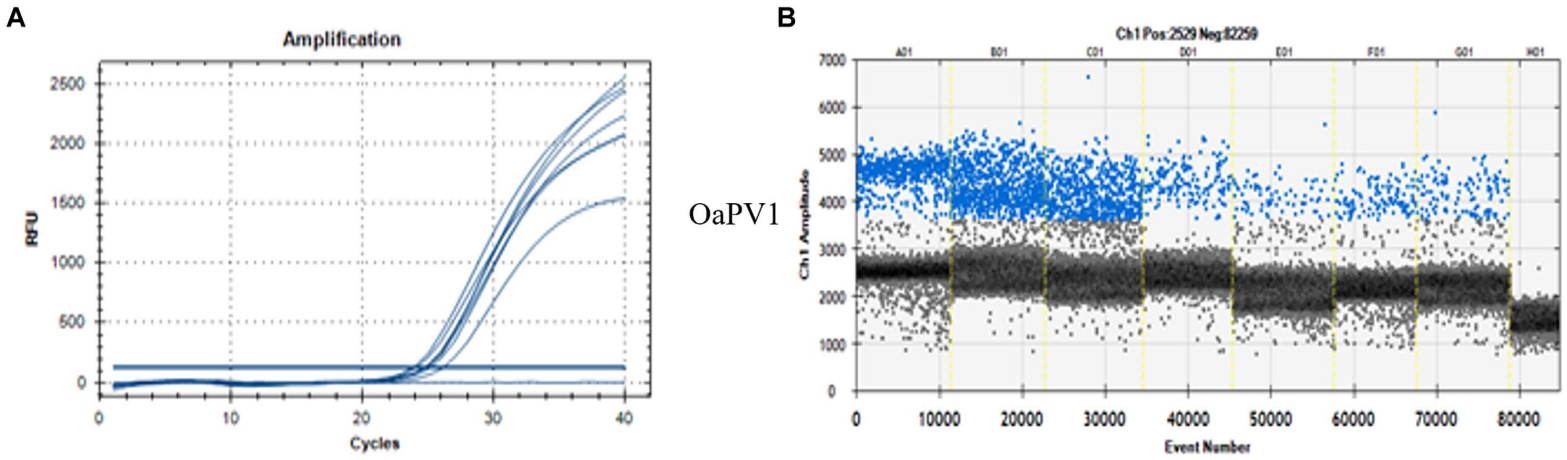
qPCR curves **(A)** and the relative rain plots of the ddPCR **(B)** for the OaPV1 horse semen DNA. For OaPV1 6 positive sample, the positive control (first line), and one negative sample (last line) are shown.

**Figure 4:**
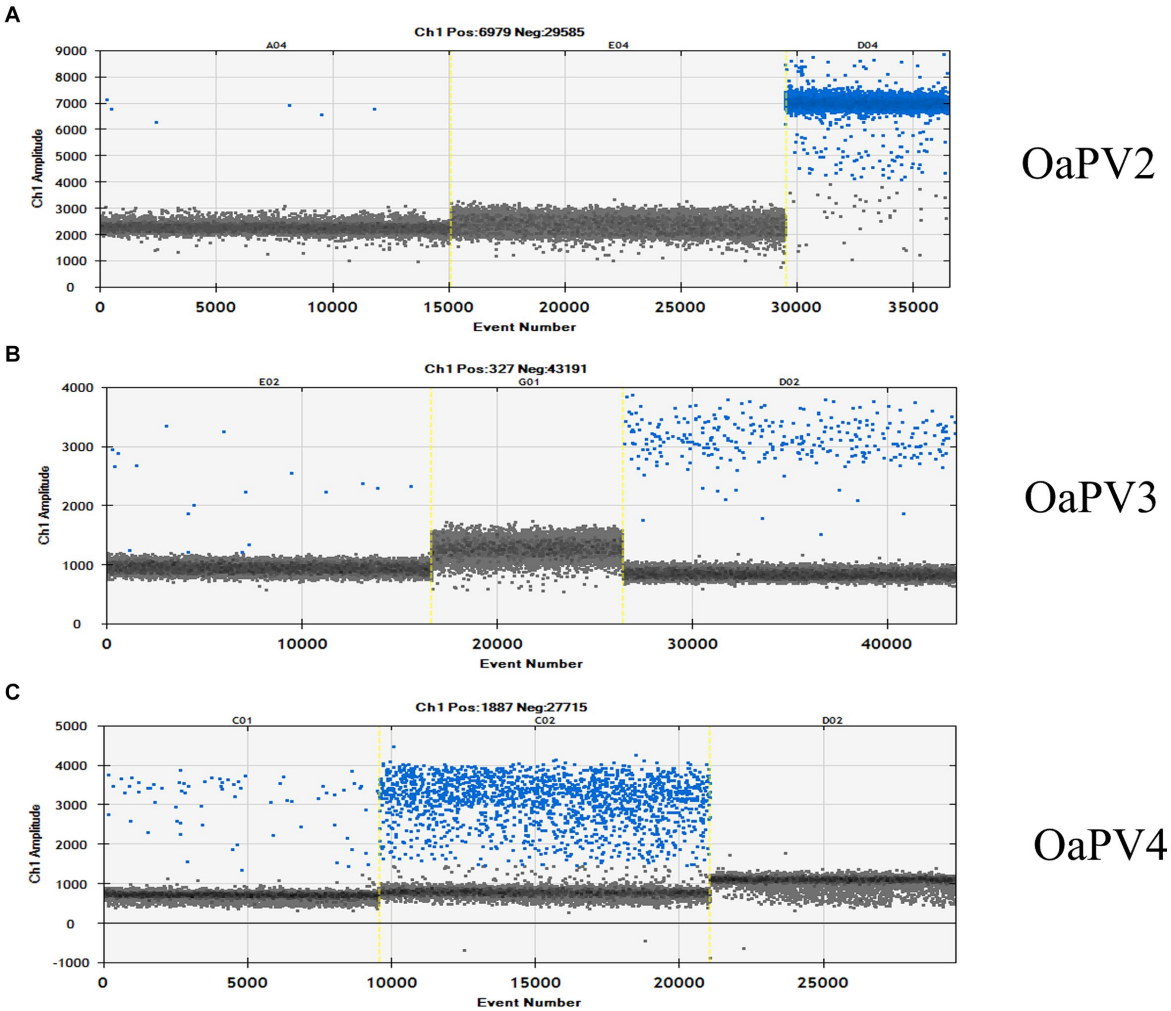
The rain plot of ddPCR for OaPV2 **(A)**, OaPV3 **(B)** and OaPV4 **(C)** detection, shown for samples that were positive and negative. QuantaSoft screenshots show the ddPCR results. A: Line A04 is a weakly positive sample; E04 is a negative sample and D04 is the positive control. B: Line E02 is a positive sample; G01 is a negative sample and D024 is the positive control. C: Line C01 is a positive sample; C02 is the positive control and D02 is a negative sample.

ddPCR detected the nucleic acids of all four OaPVs in 26 of the 27 semen samples examined (96.3%), whereas qPCR detected OaPV1 DNA only in 18 of the 27 samples (69.2%) (Figure 1). Differences between the two molecular protocols were determined to be statistically significant using McNemar’s test (*p* value ≤ 0.05). ddPCR detected a single infection in 14 semen samples (53.8%), and OaPV1 and OaPV4 genotypes were detected in nine and five samples, respectively. Furthermore, ddPCR revealed multiple infections in 12 samples (46.2%). Dual OaPV1/OaPV2 infection was seen in four samples. Double infections caused by OaPV1/OaPV3 and OaPV1/OaPV4 were detected in both semen samples. Finally, six semen samples were infected with triple infections composed of OaPV1/OaPV2/OaPV3, as revealed by ddPCR. However, qPCR failed to detect multiple infections (Figure 2). Overall, OaPV1 DNA was detected by ddPCR in 21 semen samples (80.8%) showing a very high number of copies/μL ranging from 0.5 to 9,720, while qPCR detected OaPV1 DNA in 18 semen samples (69.2%) with a low cycle threshold (CT) value thus correlating ddPCR data. OaPV1 mRNA was detected and quantified in 17 of 21 samples; its copy number varied from 6.91 to 2010 per μL. Figure 3 compares the qPCR cycle of quantification (Cq) values and ddPCR rain plots of OaPV1 in the same samples. OaPV2 DNA was detected by ddPCR in 10 semen samples (38.5%), six of them contained transcripts. OaPV3 was detected in seven semen samples (27%), two of which tested positive for mRNA. OaPV4 DNA was detected in six semen samples. OaPV4 is not transcribed (Supplementary Table S1 summarize the results). Figure 4 shows the ddPCR rain plots for semen-positive samples to OaPV2, OaPV3, and OaPV4. The number of samples positive for OaPV1 were significantly higher than the number of samples positive for OaPV2, OaPV3, and OaPV4 as demonstrated by Fisher’s test (*p* value ≤ 0.05).

Peripheral blood and semen samples of the same animals were collected from nine horses. ddPCR detected OaPV1 DNA and its transcripts in six blood samples from stallions with semen infected with active OaPV1, showing a very strong concordance. ddPCR failed to detect any nucleic acids of OaPVs in peripheral blood samples from the remaining three stallions, one of which had semen with a very low copy number of OaPV1 DNA.

**Figure 5:**
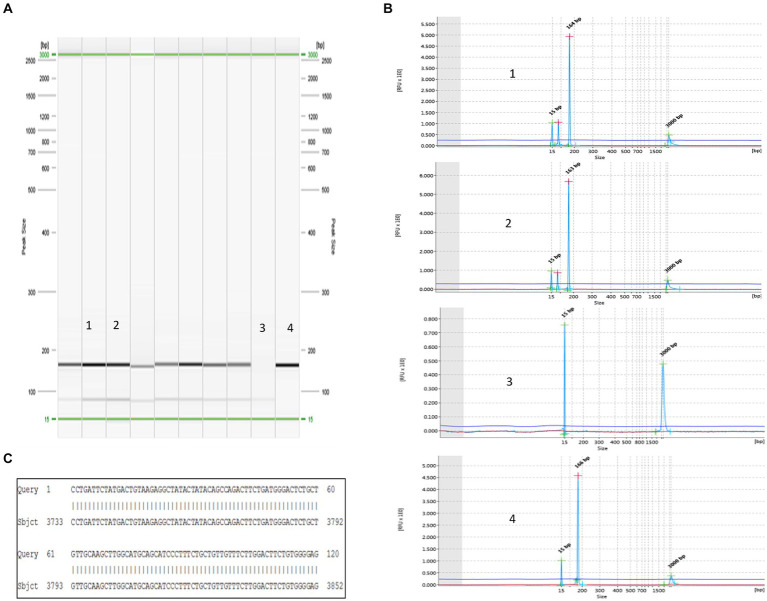
Detection of OaPV1. **(A)** Virtual gel of DNA samples from horse semen amplified for OaPV1 obtained by the QIAxcel system. In particular: lines 1 and 2: positive samples; line 3: negative control (no template) and line 4: positive control. **(B)** Visualization of OaPV1 electropherograms after electrophoretic run. **(C)** 100% identity between the sequences of the OaPV1 Seq amplicons and the sequences reported in GenBank (accession number NC_001789.1).

To validate the ddPCR and qPCR results, several positive samples containing high copy numbers of OaPV1 were subjected to type-specific standard PCR, which yielded amplicons of the expected size. These amplicons were sequenced, and subsequent BLAST alignment revealed 100% identity between the amplicon sequences and the OaPV1 E5 region (accession number: U83594.1) (Figure 5).

## 4 Discussion

We showed, for the first time, the presence of OaPV DNA and its transcripts in the semen of healthy stallions. ddPCR has been shown to be an accurate diagnostic procedure, with high specificity and sensitivity for OaPV infection. The molecular findings provided herein strengthen those of previous studies which showed that ddPCR can detect otherwise undetectable untranscribed as well as transcribed genes of papillomaviruses at very low concentrations (16, 20, 25). The OaPV2, OaPV3, and OaPV4 genotypes had a very low abundance via ddPCR, which may explain why qPCR failed to reveal their presence, thus confirming that qPCR is characterized by extreme inaccuracy in the detection of low PV viral loads. Although it is believed that the sensitivity of semen samples for PV detection is very low (27), ddPCR demonstrated a sensitivity of 96.3%, compared to that of qPCR sensitivity which was 66.7%. However, their biological significance in horse semen remains unclear. Therefore, semen from asymptomatic infected stallions may serve as an additional source of OaPVs. Stallions may have an important role in virus transmission and infection since OaPVs were found to have an evident transcriptional activity in the equine semen as documented by a very high number of mRNA copies/μL, in particular OaPV1. Our study represents a basis for further research in OaPV molecular epidemiology. It is worth noting that little information is available on the OaPV infections, which could explain the poor understanding of the biological significance of cross-species transmission of these viruses.

Recently, a great deal of interest has been given for the detection of HPV in semen, with a high percentage of men affected by unexplained infertility (28). Although a possible correlation between HPV sperm infection, asthenozoospermia, and male fertility has been suggested (29), it is believed that HPV negatively affects the fertility of both males and females (30). Therefore, HPV infection of semen may be responsible for the reduction in natural and assisted pregnancy rates and a risk factor for pregnancy interruption (31). The exact mechanism by which sperm infection impairs fertility requires further investigation (22, 30), considering that the transcriptional activity of HPV has never been detected in human semen (32).

Although numerous studies have examined the oncogenic effects of PVs, studies focusing on the impact of PVs on fertility and reproduction in large and small domestic animals are lacking. In particular, information on pregnancy outcomes in stallions with PV infection of sperm is scarce. It is worth noting that a definitive causal diagnosis could not be established in as many as 70% or more cases of pregnancy loss in mares (33), including early pregnancy loss (EPL), which occurs in approximately 8% of equine pregnancies (34, 35). While maternal factors have been recognized as important risk factors for reproductive disorders, there is no information on the importance of stallion factors in influencing the reproductive performance of mares, including EPL (34, 36). PV infection in the semen may be associated with increased incidence of equine infertility. Equine PVs have recently been proposed as possible causative agents of horse abortions (37). Similar to HPVs, OaPV infection may induce a pro-inflammatory state, thus contributing to increased inflammatory cytokines, which may have harmful consequences for reproductive parameters. Cytokines play a central role in physiological and pathological processes of the male reproductive tract (38, 39). The presence of OaPVs in the semen of normospermic horses suggests the need for prospective studies to establish the reproductive prognosis of stallions with sperm PV infections that undergo assisted reproduction techniques (ARTs). Because PV infection of semen may play a role in equine infertility, further studies are needed to understand the biological significance and epidemiology of active OaPVs in horses, as appropriate stallion semen handling is of great importance in the equine artificial insemination (AI) industry. It has been shown that HPV-positive women undergoing AI have a significantly lower clinical pregnancy rate compared to that of HPV-negative women (40). Furthermore, it has been suggested that sperm HPV infection can be a risk factor for recurrent pregnancy loss (RPL), a poorly understood early pregnancy disorder, as 60–70% of cases have no identifiable etiology (41, 42).

The mechanism by which viruses gain access to these sites remains unclear. It has been suggested that the presence of HPV DNA in the semen of asymptomatic men may be associated with HPV infections of the penile epithelium and subsequent desquamation of HPV-infected penile cells (43).

We do not exclude the possibility that OaPVs in some semen samples from asymptomatic stallions in our study may have resulted from the desquamation of OaPV-infected penile cells. However, we suggest that OaPV in equine semen mostly originates from the blood. Our findings strengthen our hypotheses. We examined nine semen and peripheral blood samples from the same stallions. We detected transcriptionally active OaPV1 in both the blood and semen of six stallions, thus showing a strong concordance of OaPV genotypes between these biological matrices and suggesting that blood may be another important source of PV infection. Peripheral blood mononuclear cells (PBMCs) of horses can transport OaPVs, which appear to be frequently transcribed, and thus represent markers of active OaPV infection (manuscript preparation). It has been shown that the peripheral blood infected with expressed papillomavirus yields infections at permissive sites with detectable viral DNA, RNA transcripts, and viral proteins (44, 45).

## 5 Conclusion

Our findings provide insights that may be useful as a starting point for further studies to understand the impact, if any, of stallion sperm PV infection on asthenozoospermia, where viral semen infection seems to be most related to the integrity of sperm DNA, successful pregnancy, and foaling, including assisted reproductive technologies (ART). Furthermore, our study clearly shows that new diagnostic approaches are needed to improve the likelihood of early diagnosis of novel pathogens that can threaten livestock health. In the current study, the conventional qPCR method was unable to detect small amounts of OaPV2, OaPV3, and OaPV4 nucleic acids, whereas ddPCR, an improved version of conventional PCR, detected and quantified DNA and its transcripts for all these genotypes. Therefore, ddPCR provides more sensitive, accurate, and reproducible detection of low-abundance pathogens and may be a better choice than qPCR for the clinical diagnosis of PV-related diseases.

## Data availability statement

The datasets presented in this study can be found in online repositories. The names of the repository/repositories and accession number(s) can be found in the article/Supplementary material.

## Ethics statement

Sperm samples were commercially purchased. Some additional samples were collected at the Didactic Veterinary University Hospital (DVUH) of Naples (protocol PG/2024/0023599, Naples University Federico II. Permission to take samples was obtained from the owners of the animals.

## Author contributions

AC: Formal analysis, Investigation, Visualization, Writing – original draft. FDF: Conceptualization, Data curation, Formal analysis, Investigation, Writing – original draft. RB: Data curation, Writing – original draft. MN: Data curation, Funding acquisition, Investigation, Writing – original draft, Writing – review & editing. GF: Data curation, Investigation, Writing – original draft. SR: Conceptualization, Supervision, Visualization, Writing – original draft, Writing – review & editing.
