# Supplementary material for: Molecular detection of transcriptionally active ovine papillomaviruses in commercial equine semen
Source: Front Vet Sci. 2024 Jul 3;11:1427370. doi: 10.3389/fvets.2024.1427370 (PMC11253197; doi:10.3389/fvets.2024.1427370)
Supplement: Supplementary file 1 [file Table_1.pdf]

Supplemental Table 1 - Nuclei acid detection and quantification of OaPVs. qPCR columns show cycle treshold (CT) numbers; ddPCR columns indicate copy numbers/µL

Horses	OaPV1 DNA		mRNA	Blood DNA	Blood mRNA	OaPV2 DNA		mRNA	Blood DNA	Blood mRNA	OaPV3 DNA		mRNA	Blood DNA	Blood mRNA	OaPV4 DNA		mRNA	Blood DNA	Blood mRNA
	qPCR	ddPCR	ddPCR	ddPCR	ddPCR	qPCR	ddPCR	ddPCR	ddPCR	ddPCR	qPCR	ddPCR	ddPCR	ddPCR	ddPCR	qPCR	ddPCR	ddPCR	ddPCR	ddPCR
1	N	1.27	58.5	27.2	3.57	N	N	N	N	N	N	N	N	N		N	N	N	N	N
2	N	3.27	92.8	21.5	114	N	N	N	N	N	N	N	N	N		N	N	N	N	N
3	N	N	N	NA		N	N	N	NA		N	N	N	NA		N	4.95	N	NA	
4	N	N	N	NA		N	N	N	NA		N	N	N	NA		N	0.37	N	NA	
5	N	N	N	NA		N	N	N	NA		N	N	N	NA		N	2.93	N	NA	
6	28.12	0.509	N	NA		N	N	N	NA		N	N	N	NA		N	1.03	N	NA	
7	N	N	N	NA		N	N	N	NA		N	N	N	NA		N	1.46	N	NA	
8	14.59	9720	2010	NA		N	0.938	N	NA		N	N	N	NA		N	N	N	NA	
9	18.18	8250	175	NA		N	1.18	N	NA		N	0.947	N	NA		N	N	N	NA	
10	20.27	7560	660	NA		N	0.747	1.89	NA		N	N	N	NA		N	N	N	NA	
11	18.47	7910	1400	NA		N	0.32	1.15	NA		N	0.913	N	NA		N	N	N	NA	
12	18.65	8540	210	NA		N	0.776	0.217	NA		N	0.363	1.65	NA		N	N	N	NA	
13	20.36	5390	590	NA		N	0.915	0,2	NA		N	0.16	0.125	NA		N	N	N	NA	
14	20.52	5070	15.6	NA		N	0.584	N	NA		N	N	N	NA		N	N	N	NA	
15	24.16	186	300	NA		N	N	N	NA		N	N	N	NA		N	N	N	NA	
16	17.35	8550	248	NA		N	0.499	0.22	NA		N	0.561	N	NA		N	N	N	NA	
17	19.93	6910	354	NA		N	0.934	0.164	NA		N	0.441	N	NA		N	N	N	NA	
18	26.12	189	N	NA		N	N		NA		N	0.359	N	NA		N	N	N	NA	
19	24.76	357	N	110	31	N	N		N		N	N		NA		N	N	N	N	N
20	24.02	541	17.6	125	8.5	N	N		N		N	N		NA		N	N	N	N	N
21	25.12	275	6.91	200	29	N	N		N		N	N		NA		N	N	N	N	N
22	24.64	349	14.4	NA		N	N		NA		N	N		NA		N	N	N	NA	
23	23.79	570	12.9	12.5	114	N	0.784	N	N		N	N		NA		N	N	N	N	N
24	24.69	331	N	NA		N	N		NA		N	N		NA		N	N	N	NA	
25		N	N	N			N		N			N		NA			N	N	N	N
26		N	N	N			N		N			N		NA			0.504	N	N	N
27		0.515	N	N			N		N			N		N			N	N	N	N

N = Negative

NA = not available
